# Supplementary figures and images for: Essential roles of Lon protease in the morpho-physiological traits of the rice pathogen Burkholderia glumae
Source: PLoS One. 2021 Sep 15;16(9):e0257257. doi: 10.1371/journal.pone.0257257 (PMC8443046; doi:10.1371/journal.pone.0257257)

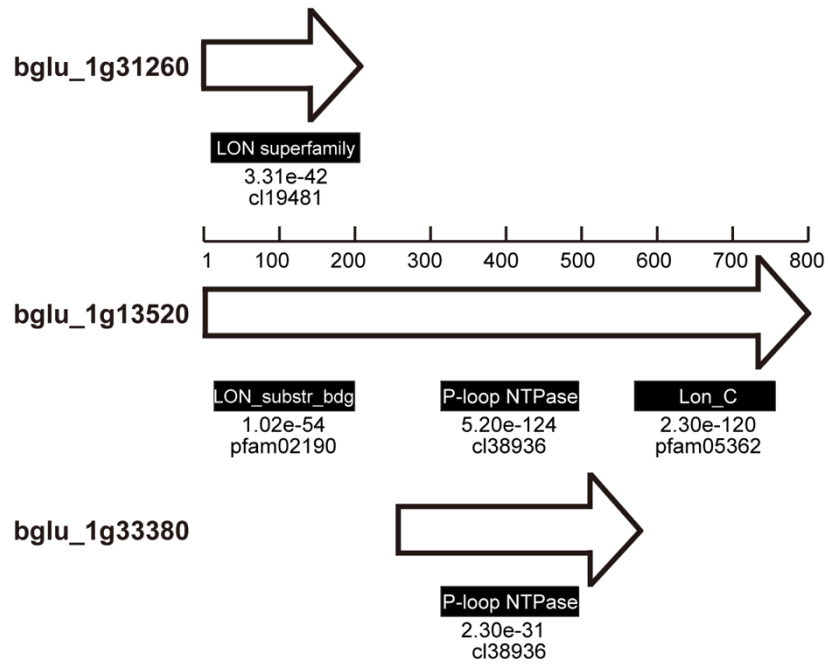

31

32 **S1 Fig. Structural alignment of three homologs of ATP-dependent Lon protease in *B.***  
33 ***glumae*.**

34

Supplement: S1 Fig — Domain names were marked in the black text boxes. The e-values and accession numbers below the representing domains were obtained from NCBI Conserved Domains. (PDF) [file pone.0257257.s001.pdf]

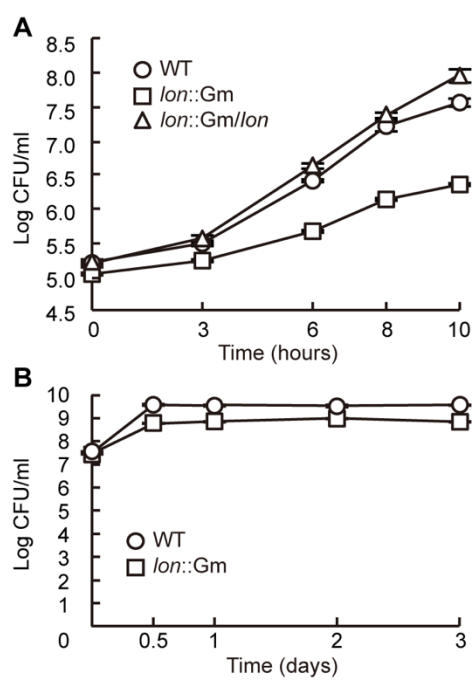

**S2 Fig. Population density of BGR1, *lon::Gm*, and *lon::Gm/lon* grown in LB supplemented with 100 mM HEPES (pH 7.0).**

Supplement: S2 Fig — (A) The lon mutant had a significantly lower initial growth rate than the wild type, even in buffered LB medium. (B) The population crash of the lon mutant was rescued after growing in LB supplemented with 100 mM HEPES (pH 7.0). (PDF) [file pone.0257257.s002.pdf]

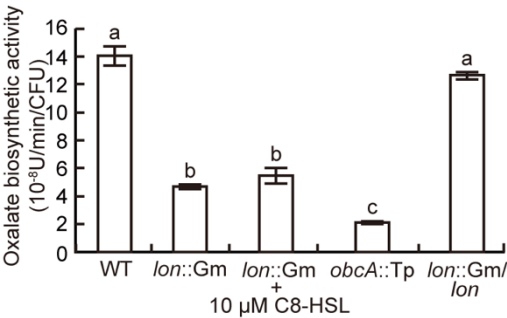

**S4 Fig. Oxalate biosynthetic component activity (units per CFU).**

Supplement: S4 Fig — The letters (a, b, and c) above each mean represent significant differences based on a one-way analysis of variance (ANOVA), followed by Tukey’s post-hoc analysis. A value of p < 0.05 indicates significant differences among strains. (PDF) [file pone.0257257.s004.pdf]

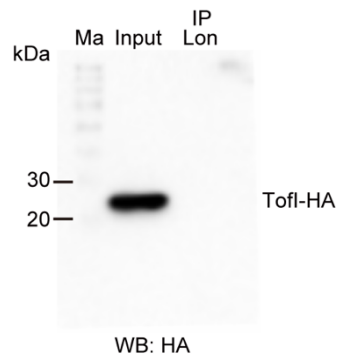

47

48 **S5 Fig. No detection of Tofl-HA in the immunoprecipitated sample using an anti-Lon**  
49 **antibody.**

50

Supplement: S5 Fig — Strain S2HA carrying the TofI-HA clone, pTOFI6, was used to determine interaction between Lon and TofI. (PDF) [file pone.0257257.s005.pdf]
