# Supplementary material for: Essential roles of Lon protease in the morpho-physiological traits of the rice pathogen Burkholderia glumae
Source: PLoS One. 2021 Sep 15;16(9):e0257257. doi: 10.1371/journal.pone.0257257 (PMC8443046; doi:10.1371/journal.pone.0257257)
Supplement: S3 Fig — Thin-layer chromatography (TLC) analysis was performed for acyl-HSLs extracted from the lon mutant (3.57 × 10^8^ CFU/mL) and the pooled lon mutant (3.57 × 10^9^ CFU/mL) grown for 24 h in LB supplemented with 100 mM HEPES (pH 7.0). The acyl-HSL sensor strain CV026 was used to visualize C6-HSL and C8-HSL. W, M, and M2 denote the wild type, lon mutant, and pooled lon mutant, respectively. (PDF) [file pone.0257257.s003.pdf]

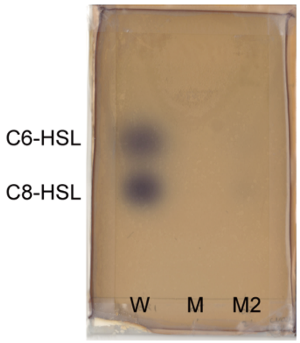

S3 Fig. Acyl-homoserine lactone (acyl-HSL) levels were lower in the *lon* mutant than the wild type, even when cells were pooled at 10^9 CFU/mL and cultured for an additional 24 h in LB supplemented with 100 mM HEPES (pH 7.0).
