# Supplementary material for: Essential roles of Lon protease in the morpho-physiological traits of the rice pathogen Burkholderia glumae
Source: PLoS One. 2021 Sep 15;16(9):e0257257. doi: 10.1371/journal.pone.0257257 (PMC8443046; doi:10.1371/journal.pone.0257257)
Supplement: S6 Fig — Comparison between ATP-dependent Lon proteases of B. glumae BGR1 (bglu_1g13520), P. aeruginosa PAO1 (AAG05192.1), P. putida KT2440 (AAN67065.1), E. coli K-12 substr. MG1655 (AAC73542.1), and A. tumefaciens C58 (WP_080866091.1). (PDF) [file pone.0257257.s006.pdf]

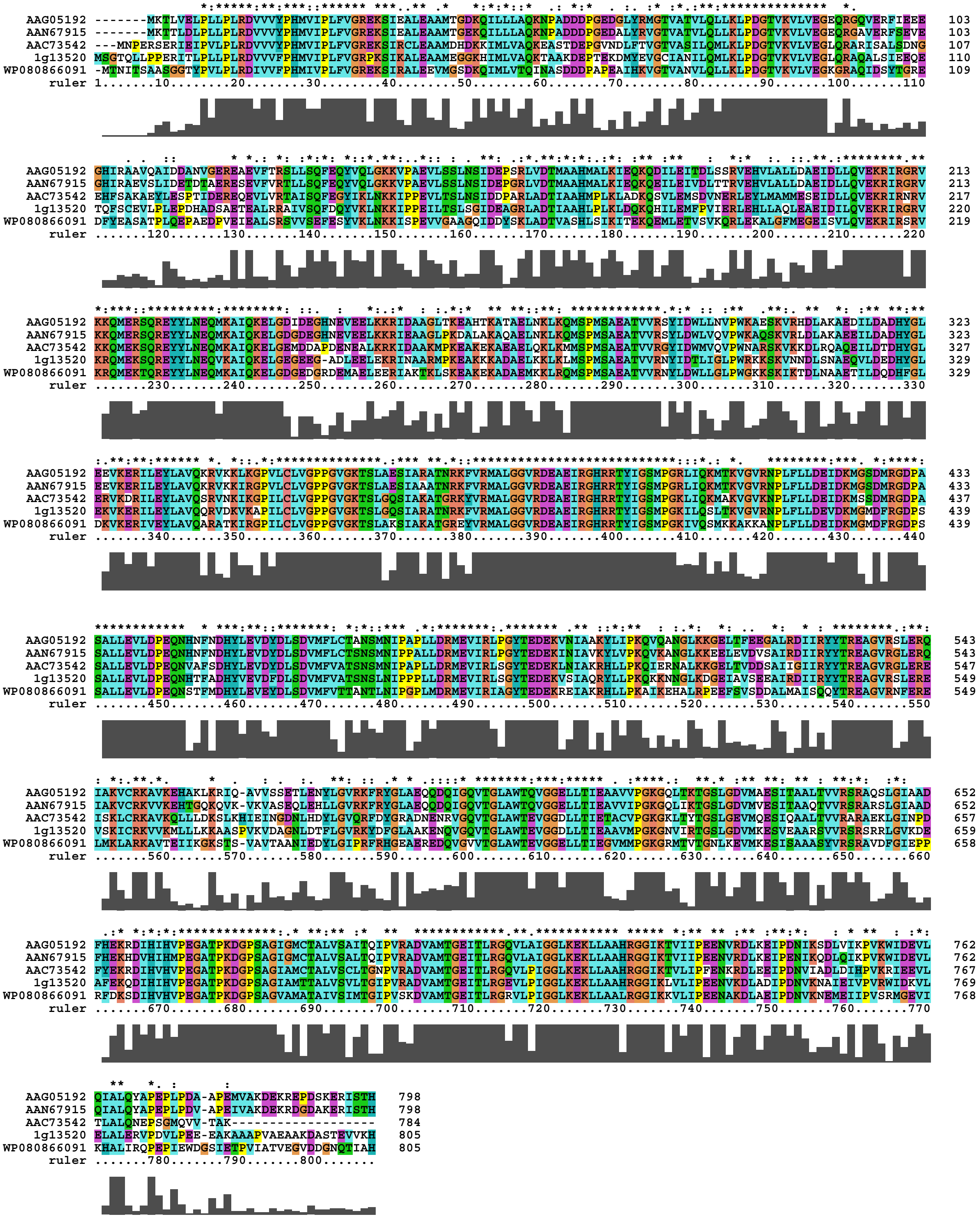

S6 Fig. Similarities among ATP-dependent Lon proteases of *B. glumae*, *Pseudomonas aeruginosa*, *Pseudomonas putida*, *Escherichia coli*, and *Agrobacterium tumefaciens*.
