# Supplementary material for: Essential roles of Lon protease in the morpho-physiological traits of the rice pathogen Burkholderia glumae
Source: PLoS One. 2021 Sep 15;16(9):e0257257. doi: 10.1371/journal.pone.0257257 (PMC8443046; doi:10.1371/journal.pone.0257257)
Supplement: S1 Table — (DOCX) [file pone.0257257.s007.docx]

**S1 Table. The strains and plasmids used in this study.**

| Strain or plasmid | Characteristics^a)^ | Source or reference |
| --- | --- | --- |
| *Burkholderia* *glumae* BGR1 | Wild type, Rif^R^ | [1] |
| BGS1 | BGR1 *tofR*::Sp | [1] |
| BGS2 | BGR1 *tofI*::Sp | [1] |
| BLONN | BGR1 *lon*::Gm | This study |
| BLONC | BGR1 *lon*::Gm/*lon* | This study |
| S2LON | BGR1 *tofI*::Sp/*lon*::Gm | This study |
| BOBCA | BGR1 *obcA*::Tp | This study |
| S2HA | BGR1 *tofI*::Sp carrying pTOFI6 | This study |
| *Escherichia coli* DH5α | F^-^ Φ80d*lacZ*Δ*M15* Δ(*lacZYA-argF*)*U169 endA1 recA1 hsdR17* (r_K_^-^m_K_^+^) *deoR thi-1 supE44 λ-gyrA96 relA* | Gibco BRL |
| BL21(DE3) | F^-^*ompT hsdS_B_*(r_B_^-^m_B_^-^)*gal dcm* (DE3) | Novagen |
| *Chromobacterium violaceum* CV026 | Autoinducer indicator strain | [2] |
| Plasmids |  |  |
| pBluescript II SK(+) | Cloning vehicle; phagemid, pUC derivative, Amp^R^ | Stratagene |
| pLAFR3 | Tra^-^, Mob^+^, RK2 replicon, Tet^R^ | [3] |
| pLa1 | 29.155 kb DNA fragment including the *lon* gene (bglu_1g13520) from strain BGR1 cloned into pLAFR3 | This study |
| pLa2 | 9.0 kb *EcoR*I-*Hind*III fragment including *lon* (bglu_1g13520) gene from pLa1 cloned into pLAFR3 | This study |
| pBS_Gm4 | PCR product of gentamycin resistant gene from pAG408 cloned into pBluescript II SK (+) | This study |
| pLa2_Gm | 0.8 kb gentamycin cassette from pBS_GM4 cloned into pLa2 *Sca*I | This study |
| pLa3 | 2.876 kb PCR fragment including *lon* (bglu_1g13520) gene and its own promoter from BGR1 cloned into pBluescript II SK(+) | This study |
| pLa4 | 2.876 kb *EcoR*I-*Hind*III fragment from pLa3 cloned into pLAFR3 | This study |
| pLa7 | 2.418 kb PCR fragment including *lon* gene from BGR1 cloned into pBluescript II SK(+) | This study |
| pLa8 | 2.418 kb *Nhe*I-*Hind*III fragment from pLa7 cloned into pET-28b | This study |
| pACYC-Duet | Plasmid for the co-expression of two target genes, Cm^R^ | Novagen |
| pTOFR4 | 0.72 kb PCR fragment including *tofR* from BGR1 cloned into pBluescript II SK(+) | This study |
| pTOFR5 | 0.72 kb *Nde*I-*Xho*I fragment from pTOFR4 cloned into pACYC-Duet | This study |
| pOBC4 | 2.716 kb PCR fragment including *obcA* and *obcB* genes and its own promoter from BGR1 cloned into pLAFR3 | This study |
| pTP2 | 0.887 kb PCR fragment including trimethoprim resistant gene cloned into pBluescript II SK(+) | This study |
| pOBC4::Tp | 0.887 kb *Kpn*I-*Xho*I fragment from pTP2 cloned into pOBC4 | This study |
| pTOFI2 | 0.697 kb PCR fragment including native promoter and *tofI* gene from BGR1 cloned into pBluescript II SK (+) | This study |
| pTOFI6 | 0.679 kb *EcoR*I-*BamH*I fragment from pTOFI2 cloned into pLAFR6 | This study |
| pRK2013 | Helper plasmid, Tra ^+^, ColE1 replicon, Km^R^ | [4] |

^a)^ Amp^R^, ampicillin resistance; Cm^R^, chloramphenicol resistance; Gm^R^, gentamycin resistance; Km^R^, kanamycin resistance; Rif^R^, rifampicin resistance; Sp^R^, spectinomycin resistance; Tet^R^, tetracycline resistance; Tp^R^, trimethoprim resistance.

**References**

1. Kim J, Kim JG, Kang Y, Jang JY, Jog GJ, Lim JY, et al. Quorum sensing and the LysR-type transcriptional activator ToxR regulate toxoflavin biosynthesis and transport in *Burkholderia glumae*. Mol Microbiol. 2004;54: 921–934. Available from: http://doi.org/10.1111/j.1365-2958.2004.04338.x

2. McClean KH, Winson MK, Fish L, Taylor A, Chhabra SR, Camara M, et al. Quorum sensing and *Chromobacterium violaceum*: exploitation of violacein production and inhibition for the detection of *N*-acyl homoserine lactones. Microbiol. 1997;143: 3703-3711. Available from: http://doi.org/10.1099/00221287-143-12-3703

3. Staskawicz B, Dahlbeck D, Keen N, Napoli C. Molecular characterization of cloned avirulence genes from race 0 and race 1 of *Pseudomonas syringae* pv. *glycinea*. J Bacteriol. 1987;169: 5789-5794. Available from: http://doi.org/10.1128/jb.169.12.5789-5794.1987

4. Figurski DH, Helinski DR. Replication of origin-containing derivative of plasmid RK2 dependent on a plasmid function provided in *trans*. Proc Natl Acad Sci USA. 1979;76: 1648-1652. Available from: http://doi.org/10.1073/pnas.76.4.1648
