# Supplementary material for: Essential roles of Lon protease in the morpho-physiological traits of the rice pathogen Burkholderia glumae
Source: PLoS One. 2021 Sep 15;16(9):e0257257. doi: 10.1371/journal.pone.0257257 (PMC8443046; doi:10.1371/journal.pone.0257257)
Supplement: S2 Table — (DOCX) [file pone.0257257.s008.docx]

**S2 Table. The primers used in this study.**

| Primer | Sequence (5’ to 3’) |
| --- | --- |
| ScaI_Gm-F | CACTTTAGTACTCGCGTCAATTCTCGAATTGACA |
| Gm_ScaI-R | CACTTTAGTACTAAGCCGATCTCGGCTTGAA |
| HindIII-La | CCAAGCTTGAAAGTGGCGGGTTTCGAATTG |
| La-EcoRI | CCGAATTCAAATCCGTCTTGTTCATCTGC |
| NheI_La | CCGCTAGCATGTCAGGCACCCAACTTCTC |
| La_HindIII | CCAAGCTTGTGCTTGACGACTTCCGTCGA |
| BamHI-obcA | AGCTAGCGGATCCGGACGGATGGGGTCCGATTTTCGG |
| obcB-EcoRI | CATGCATGAATTCTCACCGCGTCACGCGTACCAGCT |
| Tp(BamHI)-F | GACTAGGATCCCTGTCTCTTATACACATC |
| Tp-R | CTGAAGCTTGCATGCCTG |
| EcoRI_ptofI-F | GGGAATTCGATATCGCGCCGACCCTCGCGT |
| BamHI_stop_HA-tofI-R | GGGATCCTCAAGCGTAATCTGGAACATCGTATGGGTAGGCCGCTTCGGGTTGCGA |
